# Schwannoma Misdiagnosed as Adrenal Adenoma: A Case Report and Review of the Literature

**DOI:** 10.1155/2020/8020761

**Published:** 2020-01-30

**Authors:** Mussa H. AlMalki, Metib Alotaibi, Mohammad Maswood Ahmad, Muhammad Amin ur Rahman, Turki Alharthi

**Affiliations:** ^1^Obesity, Endocrine and Metabolism Center, King Fahad Medical City, Riyadh, Saudi Arabia; ^2^King Fahad Medical City, College of Medicine, King Saud Bin Abdul Aziz University for Health Science, Riyadh, Saudi Arabia; ^3^Department of Pathology and Laboratory Medicine Administration, King Fahad Medical City, Riyadh, Saudi Arabia; ^4^Department of Endocrinology, Military Hospital, Jeddah, Saudi Arabia

## Abstract

Schwannoma is a benign neurogenic tumor originating from the neural sheath of Schwann cells. It is an extremely rare cause of adrenal adenoma which is very difficult to diagnose preoperatively. We report the case of a right adrenal schwannoma discovered incidentally in a 62-year-old woman during evaluation of right flank pain. The biochemical and hormonal evaluations were unremarkable. Radiological examination revealed a 4.8 cm lesion keeping with right adrenal adenoma. Surgical intervention was done due to the large size of the tumor, and laparoscopic right adrenalectomy was performed. The postoperative course was uneventful. Histological examination established the diagnosis of schwannoma, which was further confirmed by immunohistochemical staining. In conclusion, adrenal schwannoma is extremely rare and can be misdiagnosed as nonsecreting adrenal adenoma. Complete surgical excision is the treatment of choice which is associated with favorable outcome and also helps in clarifying its histopathological nature.

## 1. Introduction

Schwannoma is a rare, benign, and slowly progressive neurogenic tumor originating from the Schwann sheath of the nerves. It occurs predominantly in the head and neck regions [1]. Visceral schwannoma in the adrenal region is extremely rare, accounting for only 0.7% of the adrenal tumors [2]. These tumors are very difficult to diagnose preoperatively, usually present as incidental findings and are often misdiagnosed as adrenal adenoma [3]. Surgery is the treatment of choice, whenever feasible. Consequently, a definitive diagnosis can only be made upon histological and immunohistochemical studies of the resected lesion [4]. Herein, we report a case of large adrenal schwannoma mimicking adrenal adenoma with a brief review of the literature.

## 2. Case Presentation

A 62-year-old Saudi woman underwent medical assessment for right flank pain. She was referred to the endocrine clinic for further evaluation. Her medical history revealed hypothyroidism on levothyroxine replacement and bilateral knee osteoarthritis, while family history was unremarkable. Clinically, she was asymptomatic with a heart rate of 71 beats/minute and blood pressure of 128/75 mmHg. Examination of her abdomen revealed no palpable mass. Blood counts and biochemical panel were within normal limits. No hormonal overproduction was detected as 24 h urinary catecholamines, plasma renin activity, plasma aldosterone concentration, and cortisol values were within normal ranges.

**Figure 1:**
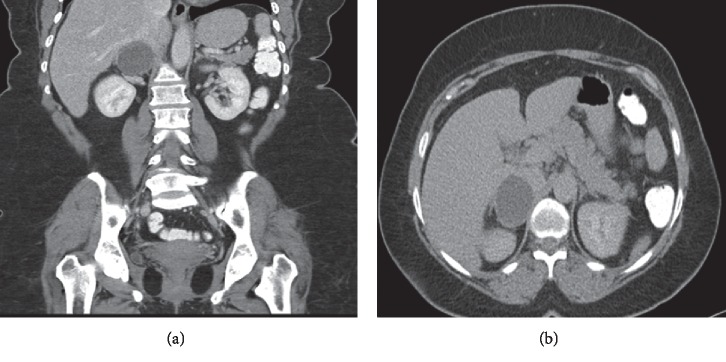
Preop CT adrenal: (a) coronal section and (b) axial section showed a 4.8 × 4.1 cm well-defined rounded right adrenal hypodense lesion. It has precontrast density of 12 HU. Its density measured 10 HU in the porto-venous phase and 15 HU on the delayed 15 minutes phase. No wall calcification. The left adrenal gland is within normal.

A computed tomography (CT) scan (Figure 1) demonstrated a well-circumscribed, uniform, hypodense fat-containing lesion 4.8 cm in diameter with a Hounsfield unit of twelve on unenhanced CT. Also, its density measured 10 HU in the porto-venous phase and 15 HU on the delayed 15 minutes phase. The absolute and relative washout value at 15 minutes of more than 55% and 35% respectively, keeping with right adrenal adenoma. Due to the large size of the tumor, surgical intervention was decided and right laparoscopic adrenalectomy was performed. The postoperative period was uneventful. On gross pathological examination, the tumor was 4.5 cm in size, encapsulated, and was present adjacent to the normal-appearing adrenal gland. A central cystic space was seen within the tumor mass.

**Figure 2:**
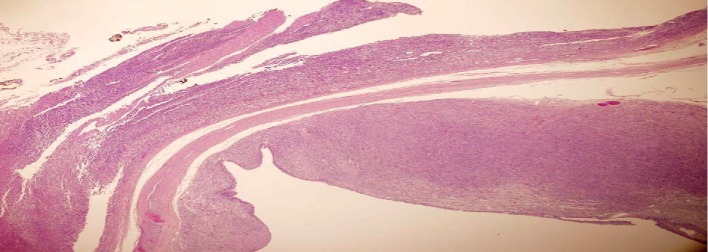
Low-power view of the adrenal gland (top) and schwannoma with capsule (middle) and cystic change (bottom). H&E ×40.

**Figure 3:**
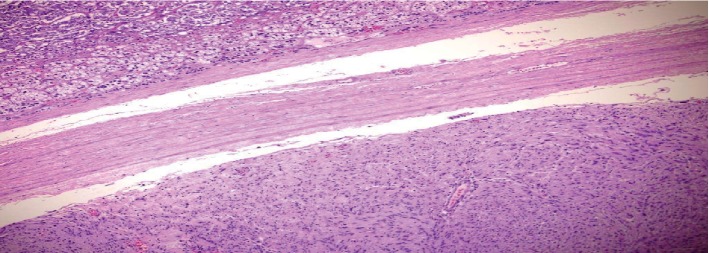
Adrenal (top), tumor capsule (middle), and schwannoma (bottom). H&E ×100.

**Figure 4:**
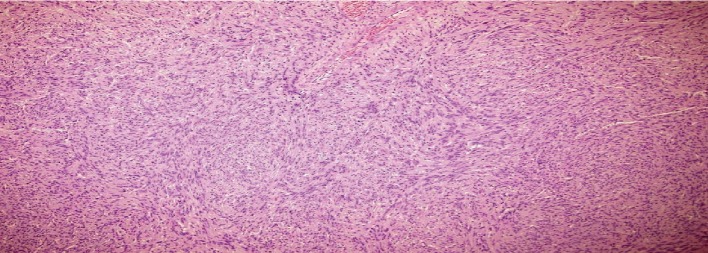
Schwannoma cell morphology, H&E ×200.

**Figure 5:**
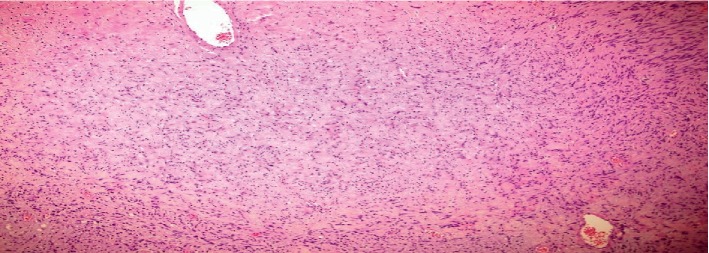
Schwannoma hypocellular areas (Antoni (B)) areas.

Histologically (Figures 2–5), the tumor comprised spindle cell proliferation arranged in small fascicles of tumor cells with elongated to wavy nuclei and scanty eosinophilic cytoplasm with indistinct cell membranes, present in a fibrillary stroma. Most of the tumor appeared very cellular with only focal alternating hypocellular (Antoni B) areas (Figure 5). The stroma also showed a variable amount of thick collagen bundles. In some areas, the tumor cell nuclei were seen palisading around a fibrillary material (Verocay bodies). Foamy and hemosiderin-laden macrophages were also seen. The tumor cells were positive with S100 on immunohistochemistry. The morphology and immunohistochemistry were consistent with a diagnosis of a schwannoma, the cellular variant which is often seen in paravertebral locations, and it shows more cellularity than the classical variant. There were no features, such as increased mitoses, pleomorphism, or necrosis, to suggest malignancy.

**Figure 6:**
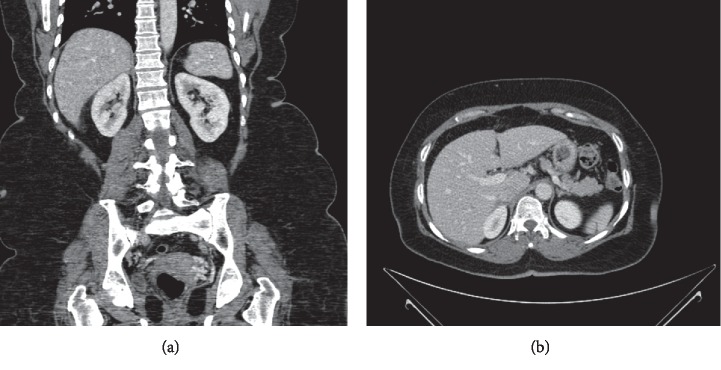
Postop CT adrenal: (a) coronal section and (b) axial section showed postresection of the right adrenal mass with no signs of residual or recurrent disease. The left adrenal gland is unremarkable.

The patient remained asymptomatic during the five-month follow-up period, and there was no evidence of any radiological recurrence or distant metastasis (Figure 6).

## 3. Discussions

Schwannoma is encapsulated, usually a benign, slow-growing tumor that arises from the Schwann cells surrounding the peripheral nerves [1]. It is usually located within the head and neck region or the peripheral extremities with lesser frequency in the retroperitoneum [5].

These tumors are rare and account only for 1–5% of retroperitoneal masses [6]. Schwannoma in the adrenal region is extremely rare, accounting for only 0.7% of the adrenal tumors with about 80 cases have been reported to date [2].

Malignant schwannoma is infrequent and typically found in the extremities and often in association with neurofibromatosis types 1 and 2 [7].

Due to their asymptomatic clinical course and nonsecreting nature, preoperatively they are often misdiagnosed as nonfunctioning adrenal adenoma.

They can occur at any age but predominantly between the second and fifth decade of life. However, retroperitoneal schwannomas are most commonly seen between 40 and 60 years of age, with a slight female predominance [7, 8]. In one series of 33 cases, a median age at the diagnosis was 49 years (14–89 years) with a slight female predominance (F : M = 1.2 : 1) [9]. Similarly, Zhou et al. also found female predilection (F : M: 1.4 : 1) with a median age of 47 years [2]. Differential diagnosis of adrenal schwannomas includes adrenal adenoma, pheochromocytoma, myelolipomas, neuroblastoma, ganglioneuroma, cortical carcinomas, cysts, and metastases [9]. Although most of the patients with adrenal schwannoma have no clinical or biochemical evidence of hormonal overproduction, a complete hormonal evaluation is still required as for other adrenal masses, including serum electrolytes, low-dose dexamethasone suppression testing, serum aldosterone renin ratio, and a 24-hour urine collection for catecholamines and metanephrines.

In one case series of 33 patients, the majority was identified incidentally, and only 13 patients had abdominal pain, flank pain, or discomfort before a specific diagnosis. One case showed elevated urinary catecholamines [9]. Recently, in case series of 31 patients by Zhou et al. the majority (84%) was identified incidentally, and only five patients had symptoms. Of these, three patients were clinically suspicious for the diagnosis of cortisol-producing adenoma; one patient with symptoms prompting the possible diagnosis of aldosterone-producing adenoma; and one patient hospitalized for acute intermittent abdominal pain without evidence of adrenal-relevant hormonal abnormalities [2].

Imaging characteristics on CT scan may aid in diagnosis. Schwannomas usually appear as a well-circumscribed, homogenous, and round or oval lesion, with slight enhancement on the CT scan with cystic degeneration or calcification [10]. In one study, it was found that the combination of necrosis with a minimal degree of tumor enhancement in the arterial phase of the CT scan increased the probability of ruling out schwannoma [11]. Furthermore, the schwannomas on MRI appear as a well-circumscribed mass with cystic degenerative changes with low signal intensity on T1-weighted images and heterogeneous high signal intensity on T2-weighted images. However, these imaging characteristics are not specific [12].

It should be noted that the preoperative diagnosis is not definitive due to limited hormonal disturbances and overlapping radiological features [13]. Therefore, it will remain unclear until after surgical intervention and histopathologic examination of the resected lesion. In our case, the clinical features and the location of the mass were strongly suggestive of an adrenal adenoma, and laboratory workup did not contribute any further other than ruling out hormonal overproduction. The final diagnosis could be uncovered only after histopathological evaluation of the resected lesion.

On gross examination, adrenal schwannomas appear as a well-circumscribed, firm, round, and tan-yellow to grayish-white mass. Although the majority of them are solid with homogenous consistency, some may have cystic areas and appear larger or may have areas of calcification or hemorrhage [14]. Histologically, schwannomas are diagnosed based on the detection of spindle cells with characteristic elongated to wavy nuclei, showing alternating cellular (Antoni A) and hypocellular (Antoni B) regions [15]. Immunohistochemically, these lesions stain positively for S100 antibodies with negative CD34, inhibin, and cytokeratin [16]. The cellular variant shows more hypercellular areas with rare to no hypocellular areas. Often there is no difficulty in histological diagnosis of schwannoma based on the characteristic morphology and S100 immunohistochemical staining.

Standard management of adrenal schwannomas is surgical resection as they are not sensitive to radiotherapy and chemotherapy [4]. The appropriate approach in adrenal schwannomas, either laparoscopic or open, remains debatable. However, Hobart et al. concluded that both techniques achieve similar results, but it remains controversial in larger schwannomas [9, 17]. Complete laparoscopic resection was achieved in our patients. The surgical histopathology consisted of spindle cell proliferation with morphological features as described above, consistent with a diagnosis of a schwannoma, cellular variant.

Prognosis is usually favorable after surgical resection. However, local recurrences have been observed in 33–50% of the cases [18]. Zhou et al. reported good survival without any evidence of recurrence or metastasis at follow-up from 7–115 months [2]. In our cases, the patient remained asymptomatic with no evidence of radiological recurrence or distant metastasis at five months after the surgery.

## 4. Conclusion

Adrenal schwannomas are rare tumors that are difficult to diagnose preoperatively. Definitive diagnosis can be made only by the histological and immunohistochemical examination of the resected lesion. Hence, complete surgical excision is the treatment of choice and is associated with a favorable outcome.
